# POTEE promotes breast cancer cell malignancy by inducing invadopodia formation through the activation of SUMOylated Rac1

**DOI:** 10.1002/1878-0261.13568

**Published:** 2023-12-27

**Authors:** Angélica Martínez‐López, Ana García‐Casas, Guiomar Infante, Mónica González‐Fernández, Nélida Salvador, Mar Lorente, Marina Mendiburu‐Eliçabe, Santiago Gonzalez‐Moreno, Pedro Villarejo‐Campos, Guillermo Velasco, Angeliki Malliri, Sonia Castillo‐Lluva

**Affiliations:** ^1^ Departamento de Bioquímica y Biología Molecular, Facultad de Ciencias Químicas Universidad Complutense de Madrid Spain; ^2^ Instituto de Investigaciones Sanitarias San Carlos (IdISSC) Madrid Spain; ^3^ Departamento de Estadística e Investigación Operativa, Facultad de Ciencias Matemáticas Universidad Complutense de Madrid Spain; ^4^ MD Anderson Cancer Center Madrid Spain; ^5^ Hospital Universitario Fundación Jiménez Díaz Madrid Spain; ^6^ Cancer Research UK Manchester Institute The University of Manchester UK

**Keywords:** breast cancer, invadopodium, POTEE, Rac1, SUMOylation, TRIO‐GEF

## Abstract

The small GTPase Rac1 (Ras‐related C3 botulinum toxin substrate 1) has been implicated in cancer progression and in the poor prognosis of various types of tumors. Rac1 SUMOylation occurs during epithelial‐mesenchymal transition (EMT), and it is required for tumor cell migration and invasion. Here we identify POTEE (POTE Ankyrin domain family member E) as a novel Rac1‐SUMO1 effector involved in breast cancer malignancy that controls invadopodium formation through the activation of Rac1‐SUMO1. POTEE activates Rac1 in the invadopodium by recruiting TRIO‐GEF (triple functional domain protein), and it induces tumor cell proliferation and metastasis *in vitro* and *in vivo*. We found that the co‐localization of POTEE with Rac1 is correlated with more aggressive breast cancer subtypes. Given its role in tumor dissemination, the leading cause of cancer‐related deaths, POTEE could represent a potential therapeutic target for these types of cancer.

## Graphical abstract

SUMOylation of the small GTPase Rac1 is essential for tumor cell dissemination. POTEE is identified as a novel Rac1‐SUMO1 effector, promoting breast cancer malignancy through invadopodium formation. POTEE activates Rac1 by recruiting TRIO‐GEF, increasing tumor cell metastasis *in vitro* and *in vivo*. Colocalization of POTEE and Rac1 correlates with aggressive breast cancer subtypes, suggesting POTEE as a potential therapeutic target.

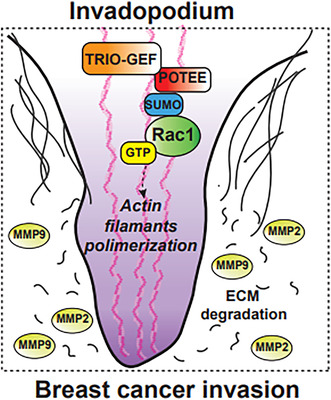

**Abbreviations**
AKT: RAC alpha serine/threonine‐protein kinase
BC: breast cancer
BM: bone marrow
CAM: chicken chorioallantoic membrane
ERK: extracellular signal‐regulated kinase
FDR: false discovery rate
GAPDH: glyceraldehyde‐3‐phosphate dehydrogenase
GFP: green fluorescent protein
GST: glutathione S‐transferase
GTP: guanosine triphosphate
HRP: horseradish peroxidase
LC–MS/MS: liquid chromatography‐mass spectrometry
mTOR: Serine/threonine‐protein kinase mTOR
NFκB: Nuclear factor NF‐kappa‐B
PBR: polybasic region
PI3K: phosphoinositide 3‐kinases
PMT: post‐traslational modification
POTEE: prostate, ovary, testes, and embryo ankyrin domain family member E
qPCR: quantitative polymerase chain reaction
Rac1: Ras‐related C3 botulinum toxin substrate 1
SUMO: Small Ubiquitin Modifier
TNBC: triple‐negative breast cancer
TRIO: Triple functional domain protein
β‐PIX: PAK‐Interacting exchange factor

## 1 Introduction

Metastasis is a multistep process in which tumor cells undergo highly complex structural and functional alterations. During this process, individual cancer cells or clusters adopt mesenchymal traits, and they detach from the primary tumor mass. This step is facilitated by the loss of E‐cadherin, a relaxation of tight junctions, and a decrease in cell–cell adhesion [1]. Once detached, the tumor cells must invade the extracellular matrix (ECM) and endothelium to spread to secondary sites. An initial step in the process of metastasis involves epithelial‐to‐mesenchymal transition (EMT), whereby neoplastic epithelial cells lose their polarity and become mobile, like mesenchymal cells, capable of invading surrounding tissues [2]. To migrate through tissues, tumor cells must degrade the ECM with proteases, mainly matrix metalloproteases (MMPs) [3]. Moreover, invasive cancer cells develop a membrane protrusion that is specialized in ECM degradation called invadopodium, a structure enriched in various proteins including actin and actin regulatory proteins, matrix‐degrading enzymes, signaling proteins, and membrane remodeling proteins [4, 5].

The Rac1 GTPase is a protein implicated in the formation of invadopodia [6, 7, 8, 9]. Rac1 is a member of the RHO family of small G‐proteins that play an important role in metastasis by enhancing tumor cell motility and invasion. Specifically, RHO proteins regulate cytoskeleton‐dependent processes during cell migration [10]. Indeed, Cdc42 and Rac1 induce integrin‐mediated cell motility and invasiveness, and activated Rac1 can regulate multiple cellular events, including cytoskeletal dynamics to maintain cell morphology, polarity, adhesion, and migration, as well as gene expression involved in cell cycle and apoptosis [11]. All these activities are important in the pathogenesis and development, and in cell dissemination or metastasis [12, 13].

RHO proteins cycle between the active GTP‐bound state and inactive GDP‐bound form, enabling them to act as molecular switches in signaling pathways. To ensure an adequate response to different extracellular stimuli, Rho GTPases are tightly controlled by three families of regulatory proteins: Rho activity can be regulated through its association with various guanine nucleotide exchange factors (GEFs) and GTPase‐activating proteins (GAPs), which control the cycling between the GDP and GTP‐bound states. Rac1 activation (GTP‐bound) is promoted by its association with GEFs, whereas its association with GAPs drives its conversion to the inactive GDP‐bound state. Moreover, Rac1 can be maintained in its inactive state through its association with Rho GDP‐dissociation inhibitor (RhoGDI) in the cytosol. Furthermore, post‐translational modifications (PTMs) of Rac1 can also regulate its activity. Modification of the carboxyl‐terminal CAAX motif in Rac1, adding either farnesyl or geranylgeranyl isoprenoid lipids, increases its hydrophobicity and facilitates its membrane localization, and hence its activation [13]. Furthermore, Ubiquitin‐like (Ubl) modifications to Rac1 in response to cell migration, including ubiquitylation and SUMOylation [14, 15], regulate the activity of Rac1. Significantly, Rac1‐SUMO1 is required to maintain Rac1 GTPase activity during cell migration required for EMT initiation [14].

In solid tumors, cells at the invasive front are primed for dissemination by the EMT, and Rac1 plays an essential role in this conversion of epithelial cells to a mesenchymal phenotype [16]. However, Rac1 is also involved in other biological processes, such as cell cycle regulation and survival, making very difficult its use as a therapeutic target. Nevertheless, Rac1 SUMOylation is only necessary for the maintenance of the activity of Rac1 required during migration/invasion but not for other events in which this GTPase participates (e.g., cell proliferation or apoptosis) [14]. Moreover, inhibiting Rac1 SUMOylation dampens the migratory and invasive behavior of breast cancer (BC) cells [17], although the mechanism by which Rac1‐SUMO1 controls cell migration is not well understood.

SUMOylation is a similar PTM as ubiquitination and neddylation, and it involves attachment of the small ubiquitin‐related modifier (SUMO) molecule to the target protein through an isopeptide bond formation. In the SUMOylation pathway, SUMO precursors are processed proteolytically at their C‐terminal dual‐glycine motifs, activated by the formation of a thioester bond through the heteromeric E1 SUMO activation enzyme (SAE1/SAE2) in an ATP and Mg^2+^‐dependent reaction, and then transferred, again via a thioester bond, to the E2 SUMO conjugation enzyme (Ubc9). SUMO is then transferred from Ubc9 to the lysine residues of substrates, either directly or with the help of E3 ligases [18, 19]. Once covalently conjugated to a protein, SUMO can regulate that protein's function by recruiting other cellular proteins, often through a non‐covalent interaction between SUMO and a SUMO‐interacting motif (SIM) in the other protein [20].

As such, we contemplated the possibility that Rac1‐SUMO1 might recruit effectors containing a SIM motif to promote cell migration. Thus, protein extracts from cells in which EMT had been induced were incubated with SUMOylated Rac1 and then analyzed by mass spectrometry (MS). As a result, POTEE (POTE ankyrin domain family member E) was identified as a new Rac1‐SUMO1 interactor in response to EMT. POTEE is one of the 12 members of the POTE family of proteins, characterized by the presence of ankyrin domains in its sequence, which are intended for protein–protein interactions. Consequently, POTEE contains six ankyrin domains in its N‐terminal region, preceded by amino acid repetitions.

In contrast to other members of the POTE family, which are commonly associated with prostate, ovary, and testis expression, POTEE is expressed widely in normal tissues [21]. However, its levels are upregulated in several tumor types, including breast, ovarian, and colon cancer, among others [21, 22, 23, 24], being considered as a cancer antigen. Consequently, POTEE has been related with poorer survival in colorectal [23, 24], pancreatic [25], and lung [26] cancer. More specifically, it has been suggested that POTEE may induce different signaling pathways to promote tumorigenesis, such as the PI3K/AKT or the SPHK1/p65 pathways [23, 25]. Indeed, POTEE is a known cancer antigen expressed in many tumors [21, 22, 23, 24]. However, its role in breast cancer remains unknown. Here we demonstrate that POTEE regulates Rac1 activity during tumor dissemination, controlling invadopodia formation through a Rac1‐SUMO1 interaction and its activation. Moreover, POTEE induces Rac1‐activation in the invadopodium by recruiting Trio‐GEF.

## 2 Materials and methods

### 2.1 Identification of proteins from Rac1‐SUMO1 by LC–MS/MS analysis and database searches

GST‐Rac1‐SUMO1 beads were incubated for 2 h at 4 °C with protein extract from MDCKII (CVCL_0424) treated cells (HGF, 10 ng·mL^−1^, for 30 min). Gel lanes from one‐dimensional (1D) gels (Nupage 4–12%) were manually cut into 20 × 1.5 mm bands using a razor blade. Processing of bands and MS analysis was performed as previously described [14]. Production data were searched against the combined forward and reverse *Canis familiaris* protein database build 2.1 downloaded from NCBI RefSeq (https://www.ncbi.nlm.nih.gov/genome/annotation_euk/Canis_lupus_familiaris/105/) using the same criteria as previously described [14]. These criteria resulted in a false discovery rate (FDR) of 0.5% at the protein level for this data set. MDCKII cell line was obtained from Angeliki Malliri (Manchester University).

### 2.2 Reagents

EHop‐016 (#SML0526‐5MG), Crystal Violet (#C3886), 2′,3′,4′‐trihydroxy‐flavone, 2‐(2,3,4‐trihydroxyphenyl)‐4H‐1‐Benzopyran‐4‐one (2‐D08; #SML1052), Phorbol 12, 13‐dibutyrate (PBDu; #P1269), were all purchased from MERCK (Darmstadt, Germany).

### 2.3 Cell culture

The human cell lines MDA‐MB‐231 (RRID: CVCL_0062), HCC‐1569 (RRID: CVCL_1255) and HEK293T (RRID: CVCL_0063) were purchased from the American Type Culture Collection (Manassas, VA). The cell lines, included MDCKII, were grown in complete RPMI or DMEM containing 4.5 g·L^−1^ glucose and l‐glutamine (Sigma Aldrich, St Louis, MO, USA), and supplemented with 56 IU·mL^−1^ penicillin, 56 mg·L^−1^ streptomycin (Invitrogen, Carlsbad, CA) and 10% fetal bovine serum (FBS, #16sV30180.03: LINUS). The cells were maintained at 37 °C in a humid atmosphere containing 5% CO_2_ and they were routinely tested for mycoplasma contamination by PCR (F‐GGCGAATGGGTGAGTAACA and R‐CGGATAACGCTTGCGACCT). The cell lines were analyzed for authentication at the Genomics Core Facility of the Instituto de Investigaciones Biomédicas “Alberto Sols” (CSIC‐UAM, Madrid) in the past 3 years using STR PROFILE DATA, the STR amplification kit (GenePrintR 10 System, Promega, Madison, WI, USA), STR profile analysis software genemapper ^®^ v3.7 (Life Technologies, Carlsbad, CA, USA) and a Genomic Analyzer System ABI 3130 XL (Applied Biosystems, Carlsbad, CA, USA).

### 2.4 Transfection experiments

Plasmid transfection was carried out with 5 μg of the vectors: pEGFP‐Rac1 wild type (WT), pEGFP‐Rac1∆CT (∆CT), pEGFP‐POTEE, pEGFP‐C3 (empty vector), pGlo‐Myc or Myc‐POTEE (Origene #RC218458, Carlsbad, CA, USA). The pEGFP‐POTEE vector was obtained by subcloning Myc‐POTEE into the pEGFP‐C1 vector using the XhoI and BamHI restriction enzymes (Thermo Scientific, Waltham, MA, USA). The pEGFP‐TRIO or pEGFP‐TRIO dominant negative (DN) vectors were generated as described in [6]. Cells were transfected using Lipofectamine 2000 (Invitrogen, #11668‐027) and incubated for 24 h before starting the experiments. The IVA cloning system [27] was used to clone the GFP‐Rac1ΔCT construct, amplified from the pEGFP‐Rac1 WT plasmid using the following primers: Fw ∆CT: CTCAAGACAGTGTTTGACGAATAAGAATTCTGCAGTCGACGGTACCG; Rv ∆CT TTCGTCAAACACTGTCTTGAGTCCTCG.

### 2.5 Protein analysis

Proteins were analyzed by western blots as described previously [28]. In brief, proteins were extracted from the tumors in FISH buffer [Glycerol 100X, 1 m Tris (pH 7.4), 5 m NaCl, 1% v/v NP40 and 1 m MgCl_2_] or from the cell lines with TNES buffer [100 mm NaCl, 1% v/v NP40, 50 mm Tris–HCl (pH 7.6), 20 mm EDTA], both containing protease and phosphatase inhibitor cocktails (Thermo Scientific #15672129). The proteins recovered were quantified using a Bradford Protein Assay (Bio‐Rad #5000006, Berkeley, CA, USA), resolved by SDS/PAGE on 10 or 12% gels (Bio‐Rad #456‐8085) and transferred to polyvinylidene difluoride (PVDF) membranes (Immobilon‐P, Millipore, Burlington, MA, USA). The membranes were then probed with the primary antibodies raised against: Rac1 (1 : 1000, clone 102, #610651: BD Biosciences, Franklin Lakes, NJ, USA), POTEE (1 : 500, #ab108190: Abcam, Cambridge, UK), Cyclin D1 (1 : 1000, #sc8396: Santa Cruz Biotechnology, Heidelberg, Germany), Tubulin (1 : 1000, DM1A, #T6199: Sigma‐Aldrich), Actin (1 : 1000, #A5441: Sigma‐Aldrich), GAPDH (1 : 1000, #G8795: Sigma‐Aldrich), GFP (1 : 1000, #SAB4301795: Sigma‐Aldrich), HSP90 (1 : 1000, #515081: Santa Cruz Biotechnology), Laminin (1 : 1000, #7292: Santa Cruz Biotechnology), Cortactin (1 : 1000, 4F11, #ab33333: abcam), Phospho AKT‐Ser473 (1 : 1000, 193H12, #4058: Cell Signaling), total AKT (#9272: Cell Signaling, Danvers, MA, USA), Phospho‐ERK1/2‐Thr202/Tyr204 (1 : 1000, #9101: Cell Signaling), total ERK1/2 (1 : 1000, L34F12, #4696: Cell Signaling). Antibody binding was detected with horseradish peroxidase (HRP)‐conjugated anti‐mouse, anti‐rabbit, or anti‐sheep secondary antibodies (1 : 10 000: Bio‐Rad), and visualized by enhanced chemiluminescence (ECL, #170‐5061: Bio‐Rad). The images were obtained with the ImageQuant LAS 500 chemiluminescence CCD camera (GE Healthcare Life Sciences, Chicago, IL).

### 2.6 Cell viability

MDA‐MB‐231 cells were plated for 24, 48, and 72 h, at which point they were fixed for 10 min in PBS with 4% paraformaldehyde (PFA) at room temperature (RT) and then stored in PBS with 0.05% azide at 4 °C prior to crystal violet staining for 30 min at RT. The plates were washes with tap water and then with 1% Triton X‐100 in PBS to remove the crystal violet from the cells before measuring their OD at 570 nm.

### 2.7 Rac1 GTPase assay

The measurement of Rac1 activity has been previously described [29, 30]. Briefly, the endogenous active GTP‐bound Rac1 was measured using a GST‐PAK1 binding domain (PBD) pull‐down assay. The cells were lysed in FISH buffer [Glycerol 100X, 1 m Tris (pH 7.4), 5 m NaCl, 1% (v/v) NP40 and 1 m MgCl_2_] and equal volumes were incubated for 15 min at 4 °C with GST‐PBD beads (20 μg). The complexes precipitated were washed three times with excess lysis buffer, and after the final wash, the supernatant was discarded and the beads were suspended in 5X Laemmli sample buffer (10 μL). Active Rac1 was detected in western blots and the amount of GTP‐bound Rac1 was normalized to the total amount of this GTPase in cell lysates for each sample.

### 2.8 Subcellular fractionation

Cells were washed and treated for 5 min with ice‐cold hypotonic lysis buffer containing protease and phosphatase inhibitor cocktails (Thermo Scientific #15672129): [10 mm Tris–HCl (pH 7.4), 1.5 mm magnesium chloride, 5 mm potassium chloride, 1 mm DTT, 0.2 mm sodium vanadate, 1 mm PMSF (phenylmethanesulfonylfluoride)]. The lysates were homogenized by sonication at low speed= and then centrifuged at 700 ***g*** for 3 min to pellet the nuclei and intact cells. The supernatants were then spin at 40 000 ***g*** for 30 min at 4 °C and the supernatant containing the cytosol was recovered. The crude membrane pellet was then washed gently with hypotonic lysis buffer, and equal amounts of protein from the membrane and cytosol fractions were then analyzed in western blots.

### 2.9 Boyden chamber cell migration/invasion assay

Cell migration was assayed in Boyden chambers (8.0 μm pore‐size polyethylene terephthalate membrane with a cell‐culture insert: VWR, Radnor, PA; #VWRI734‐2744) as previously described [30]. Briefly, the cells were trypsinized and counted, and cell suspensions containing 5 × 10^4^–1 × 10^5^ cells in 200 μL of serum‐free medium were added to the upper chamber, with 500 μL of the appropriate medium added to the lower chamber. The transwell inserts were incubated at 37 °C for 24 h with MDA‐MB‐231 cells, or 48 h with HCC‐1569 or T‐47D, after which the cells on the inside of the transwell inserts were removed with a cotton swab, while those on the underside of the insert were fixed and stained. Photographs were taken of five random fields per insert and the cells were counted to calculate the proportion of cells that had transmigrated. Boyden chambers with 0.7 mg·mL^−1^ of matrigel (Cell Biolabs, San Diego, CA; #CBA‐110) and 1.5 × 10^5^ cells were used to assay cell invasion. MDA‐MB‐231 were exposed to EHop‐016 (2 μm) or 2D08 (20 μm) for 24 h, that selectively inhibits this GTPase and SUMOylation, respectively.

### 2.10 Gelatine zymograms

The gelatine zymograms were analyzed as previously described [30, 31]. Briefly, to assess the MMP‐2 and MMP‐9 metalloprotease activity, the medium conditioned (CM) by MDA‐MB‐231 was collected. The protein concentration was determined by the Bradford method and the proteolytic activity in the CM that contained 40 μg of protein was assayed on gelatin gels for each condition. Briefly, samples were mixed with non‐reducing buffer containing 2.5% SDS and separated in 8% acrylamide gels co‐polymerized with gelatin (1 mg·mL^−1^), as described previously [32]. After electrophoresis at 72 V for 2.5 h, the gels were rinsed twice in 2.5% Triton X‐100 and then incubated at 37 °C for 24 h in activation MMPs buffer [5 mm CaCl_2_,50 mm Tris–HCl (pH 7.4) for MMP‐2 or (pH 8.3) for MMP‐9]. The gels were fixed and stained with 0.25% Coomassie Brilliant Blue G‐250 in 10% acetic acid and 30% methanol. Proteolytic activity was visualized through the clear bands that appeared against the background stain of undigested substrate in the gel and it was quantified using ImageJ software (NIH, Bethesda, MD).

### 2.11 Invadopodia formation assays

Breast cancer cell lines were plated on coverslips that were pre‐treated for invadopodia formation assays, following the protocol described [33]. To prepare the coverslips, they were incubated overnight with 20% sulfuric acid and sterilized using 96% ethanol. Then, a solution of 50 μg·mL^−1^ poly‐l‐Lysine diluted in PBS was applied to the coverslips, followed by treatment with 0.5% glutaraldehyde in PBS. The coverslips were then incubated with fluorescent gelatin proportion 1 : 25 mix of gelatin from pig skin Oregon green 488 conjugate (Thermo scientific, G13186) and 0.2% gelatin from bovine skin (Sigma, G1393) at 37 °C for 30 min. MDA‐MB‐231 and HCC‐1669 cells were cultured in serum‐supplemented medium for 24 h. The cells grown on the coverslips were fixed and permeabilized using a solution of 4% formaldehyde (PFA) and 0.5% Triton X‐100 in PBS. Blocking was performed using 3% BSA, and then the cells were incubated with phalloidin‐TRITC (Phalloidin‐iFluor 594, #ab176757: Abcam) for 30 min at 37 °C. Finally, the coverslips were mounted onto glass slides with DAPI using the Gold antifade reagent (Invitrogen; #P36935). Immunofluorescence analysis was conducted using a 100 × immersion objective and visualized with a Zeiss microscope (Zeiss Axioplan 2). Images were analyzed with image j software, version 1.44p (NIH).

### 2.12 Immunofluorescence microscopy

The immunostaining was analyzed as previously described [30]. Briefly, cells were plated and grown on glass coverslips for 48 h, fixed for 10 min with 4% PFA at RT and then stored in PBS with 0.05% sodium azide at 4 °C until they were immunostained. Briefly, the cells were permeabilized for 3 min at 4 °C in PBS containing 0.5% Triton X‐100 and the coverslips were then probed overnight at 4 °C with the primary antibody against Ki67 (1 : 200, #PA1‐21520: Thermo Scientific), POTEE (1 : 500, #SAB1301635: Sigma‐Aldrich), Rac1 (1 : 200, #610650: BD Biosciences), Phalloidin (Phalloidin‐iFluor 594, #ab176757: Abcam), Cortactin (1 : 200, #ab33333: Abcam) or green fluorescent protein (GFP, 1 : 1000, #MA5‐15256: Thermo Scientific). After rinsing thoroughly, the coverslips were incubated with Alexa Fluor 488, 594, or 635 goat anti‐mouse‐IgG or anti‐rabbit‐IgG secondary antibodies (Invitrogen), counterstained with DAPI and then mounted on glass slides using the Gold antifade reagent (#P36935, Invitrogen). Immunofluorescence was analyzed under a Zeiss microscope (Zeiss Axioplan 2) and to calculate the proliferation ratio using the Ki67 marker, we counted the total number of positively stained tumor cells in each image/field and the total number of tumor cells in each image. The percentage of Ki67^+^ cells was calculated as: No. of positive tumor cells/total No. of tumor cells × 100.

### 2.13 Human tumor samples

The Kaplan–Meier (KM) plotter free database was used to evaluate how POTEE overexpression is associated with the evolution of BC patients [34]. Breast tumor studies were performed with the approval of The Bioethics Committee at the MD Anderson Cancer Center from Madrid (License number MD21/004) in accordance with the tenets of the Helsinki Declaration. Written informed consent was obtained from each patient before POTEE and Rac1 expression was evaluated in samples from different molecular subtypes of human tumors by immunofluorescence (IF). All samples were collected before 2021. IF staining was performed following the protocol described previously [35], and POTEE, Rac1 or POTEE‐Rac1 co‐localization was analyzed. Quantification of co‐localization between POTEE and Rac1 in the tumor samples was performed by analyzing five random areas. The number of cells showing co‐localization was divided by the total number of cells (identified by DAPI staining). This method allowed us to calculate the percentage of co‐localization for each tumor sample. Similarly, we quantified the individual expression of Rac1 or POTEE. We analyzed four different breast cancer tissues for each molecular subtype, with a total of four samples per subtype (*N* = 4). Immunofluorescence was analyzed under a Leica microscope (Leica SP8 Confocal Laser Scanning Microscope, with Dmi8 stand).

### 2.14 Chicken embryo experiments

*In vivo* experiments using chicken embryos do not require any special permits as long as the embryos are sacrificed before hatching, as occurred in this study. For the chorioallantoic membrane (CAM) xenografts, premium specific pathogen‐free (SPF), fertile, day 11 fertilized chicken eggs were inoculated with 2 × 10^6^ MDA‐MB‐231 GFP^+^ cells expressing Myc‐tag or Myc‐POTEE. The tumor cells were injected into the chicken embryo, placing them in the middle of a small sterile plastic ring that prevented their diffusion. After 7 days growth, the tumors and bone marrow were excised, weighed, and measured, and immediately frozen for protein and qPCR analysis.

### 2.15 Quantitative detection of human tumor cell metastasis

Human tumor cells were detected by quantifying human Alu sequences in chicken bone marrow (BM) DNA extracts, a modification of the method developed previously [36]. Briefly, the BM was snap frozen in liquid nitrogen and the genomic DNA extracted using proteinase K, analyzing the genomic DNA in qPCR reactions. Human cells in the chicken tissues were detected using primers specific for the human *Alu* repeat sequences (F‐ACGCCTGTAATCCCAGCACTT; and R‐TCGCCCAGGCTGGAGTGCA) present in the genomic DNA extracted from the chicken tissues. Amplification of chicken *Gapdh* (*chGapdh*: F‐ACTGGGAGGTAGAGACTGGC and R‐CAAGGATCTGTGTTGCCATGC) was used as an internal control for the total amount of tissue.

### 2.16 Statistical analyses

Unless otherwise indicated, the data are expressed as the mean ± standard error of the mean (SEM) or standard deviation (SD) and two groups were compared with a Mann–Whitney *U* test (non‐parametric) or *t*‐test (parametric). More than two groups were compared with a Kruskal–Wallis test (followed by Dunn's multiple comparison *post‐hoc* test) or ANOVA (followed by Turkey's multiple comparisons test). The proportion of positive cells was evaluated using the *χ* ^2^ test and the tumor volumes in the chicken were compared using a multiple *t*‐test. For all the analyses, *P*‐values ≤ 0.05 was considered statistically significant.

## 3 Results

### 3.1 POTEE interacts with Rac1‐SUMO1 during cell migration

**Fig. 1:**
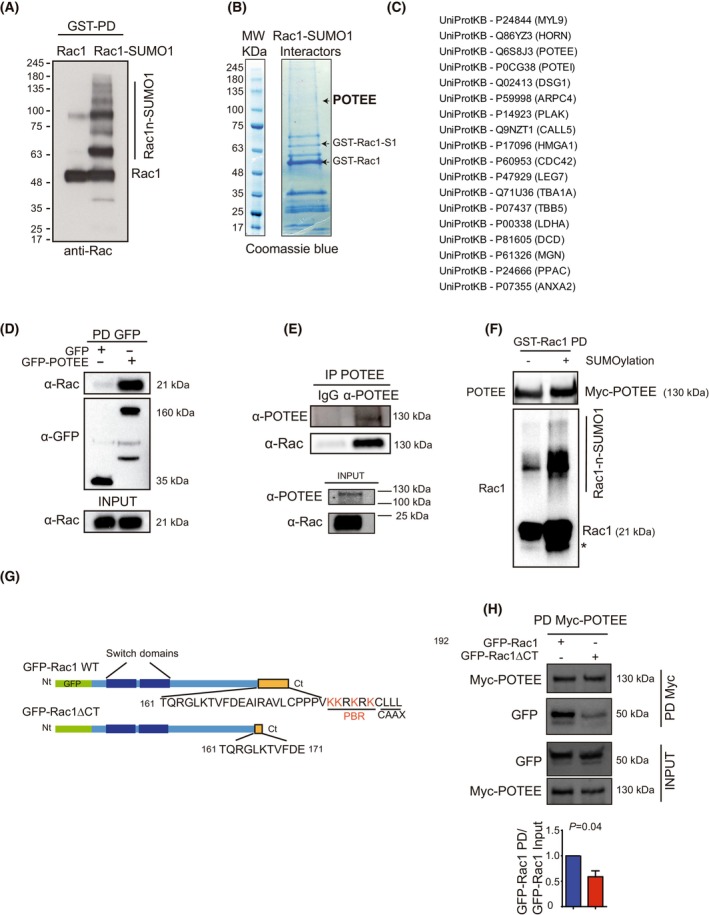
POTEE interacts with Rac1 in response to migration (A) GST–Rac1 was incubated *in vitro* in the presence or absence of the complete SUMOylation assay components, resolved by SDS/PAGE and assayed in western blots. (B) The GST–Rac1–SUMO1 reaction product was incubated with a protein extract of MDCKII cells exposed to hepatocyte growth factor (HGF, 10 ng·mL^−1^), and this mixture was resolved by SDS/PAGE and Coomassie stained. (C) The bands obtained in a GST‐Rac1‐SUMO1 pull‐down assay were excised and subjected to in‐gel trypsin digestion. The resulting peptides were analyzed by MS, and MaxQuant data processing was employed to detect modified and unmodified peptides. The list of proteins identified by MS is provided with the UniProtKB identification numbers. (D) GFP epitope‐tagged POTEE was expressed in HEK293T cells and the proteins recovered by GFP immunoprecipitation were detected in western blots. (E) Rac1 immunoprecipitates with endogenous POTEE from MDA‐MB‐231 cells was analyzed by western blots. (F) The GST–Rac1–SUMO1 reaction was carried out with a protein extract from MDA‐MB‐231 cells transfected with Myc‐POTEE. (G) Representative scheme of the Rac1 forms used in the experiments. GFP (green) is fused to the N‐terminal (Nt) of the Rac1 protein (light blue bar). Removing the C‐terminal (Ct) of Rac1, which includes the PBR and CAAX motifs along with the previous residues results in the GFP‐Rac1ΔCT form. (H) MDA‐MB‐231 cells transfected with the GFP‐Rac1WT or GFP‐Rac1∆CT and Myc‐POTEE plasmids. The POTEE immunoprecipitated with GFP was detected in western blots. Data presented are shown as mean ± SEM. The results represent three independent experiments. Significant differences between two groups were assessed using a *t*‐test and considered significant when *P* < 0.05.

To identify proteins that interact with Rac1‐SUMO1 during cell migration, we performed an *in vitro* GST‐Rac1 SUMOylation assay, pulling down the GST‐Rac1‐SUMO1 modified protein (Fig. 1A). The SUMOylated Rac1 protein was incubated for 2 h with native protein complexes from MDCKII (Madin‐Darby canine kidney strain II) cells after they had been treated with hepatocyte growth factor (HGF), a ligand for the c‐Met growth factor receptor and a stimulator of migration (promoting the EMT). This assay was performed when Rac1 was activated and the cells had begun to migrate. The protein complexes purified were analyzed by liquid chromatography‐mass spectrometry (LC–MS/MS) (Fig. 1B), and among the known and putative Rac1 interacting proteins in the HGF stimulated cell extracts that bound to Rac1‐SUMO1, we identified a novel Rac1 interacting protein, POTEE (Fig. 1C). We confirmed that exogenous and endogenous POTEE interacts with Rac1 (Fig. 1D,E). Moreover, Rac1‐SUMO1 appears to interact with POTEE more efficiently (Fig. 1F). Rac1 can be SUMOylated at any of the lysines within the C‐terminal polybasic region (PBR) [14] and as expected, deletion of the Rac1 C‐terminal domain (GFP‐Rac1∆CT) impaired its interaction with POTEE (Fig. 1G,H).

Together, these observations suggest that POTEE is a novel Rac1‐SUMO1 effector that interacts with Rac1 when cells undergo EMT.

### 3.2 POTEE localizes at the membrane ruffles where it influences cell migration through Rac1 activation.

**Fig. 2:**
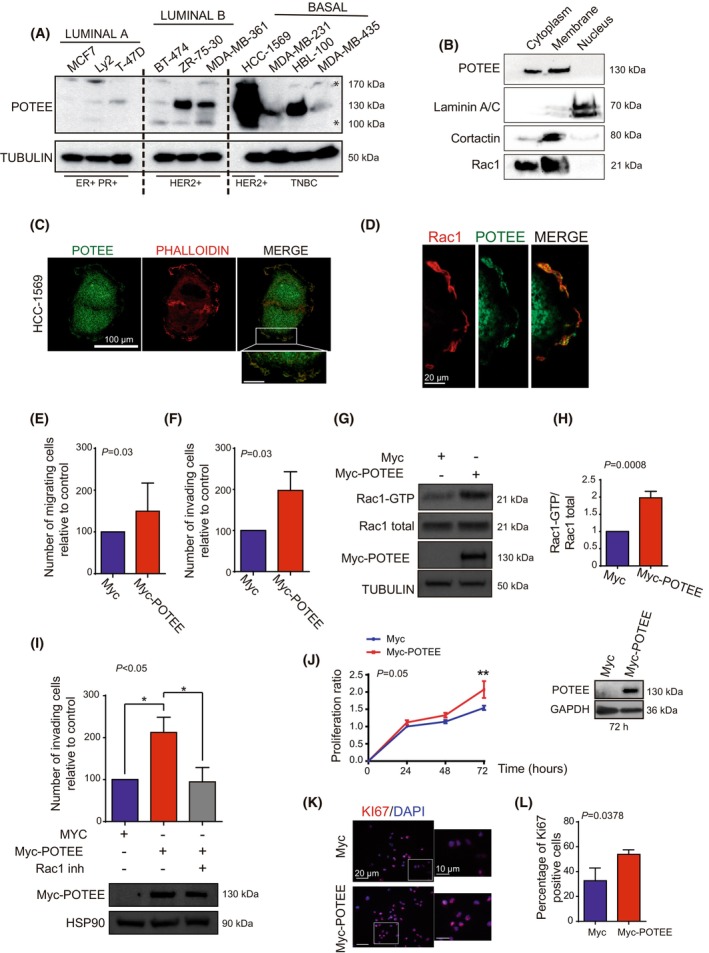
High levels of POTEE increase the tumorigenic capacity of breast cancer cell lines by activating Rac1 (A) POTEE protein expression in a panel of cell lines representing different breast cancer subtypes. Asterisk refers to non‐specific bands. At the top of the image, the molecular subtype is indicated, and at the bottom, the expression of the main breast cancer markers is shown. (B) The HCC‐1569 HER2+ cell line was fractionated and the proteins recovered were assayed in western blots. (C) Representative images of endogenous POTEE and phalloidin localization in HCC‐1569 cells. Scale bar, 100 μm. (D) Localization of endogenous POTEE and Rac1 in HCC‐1569 cell membrane ruffles. Scale bar, 20 μm. (E) Transwell migration assays showing the number of migrating MDA‐MB‐231 breast cancer cells overexpressing POTEE relative to the controls (*t*‐test) The results are representative of three independent experiments mean ± SEM. (F) Matrigel invasion assay showing the number of invading MDA‐MB‐231 cells relative to that induced by the controls (*t*‐test). Data are representative of three independent experiments mean ± SEM. (G) Representative western blot of MDA‐MB‐231 cells probed for active Rac1 in response to POTEE overexpression. (H) Quantification of the relative normalized amounts of Rac1–GTP in G, as determined by scanning densitometry using imagej software. Data from three independent experiments are presented as mean ± SEM, and statistical analysis was performed using a *t*‐test. (I) Matrigel invasion assay showing the number of invading MDA‐MB‐231 cells in the presence or absence of the Rac1 inhibitor (2 μm) relative to that induced by the control (ANOVA test; Tukey's *post‐hoc* test), with a representative blot of the cell transfection shown. The results are representative of three independent experiments mean ± SEM. (J) A representative western blot of MDA‐MB‐231 cells transfected with Myc‐POTEE, the cell viability of which was assessed by crystal violet staining. The results are representative of three independent experiments mean ± SEM. (K) Representative image of the immunofluorescent evaluation of Ki‐67 expression in MDA‐MB‐231 cells and (L) the relative proportion of Ki‐67^+^ cells (*χ* ^2^ test). (scale bar, 10 μm). The results represent three independent experiments presented as mean ± SEM. Significant differences were assessed and considered when *P < 0.05. When *P* > 0.05, differences were considered non‐significant (ns).

To investigate the role of POTEE in breast cancer (BC), the expression of this protein was analyzed in a panel of cell lines representing different BC subtypes (Fig. 2A). POTEE protein was particularly intense in the more aggressive luminal B‐HER2+ and TNBC subtypes. Additionally, an increase in the SUMOylation state of Rac1 was observed in the different BC molecular subtypes (Fig. S1A). Furthermore, in fractionation experiments performed on the HER2‐positive HCC‐1569 breast cancer cells, POTEE clearly accumulated in both the membrane and cytoplasmic fractions (Fig. 2B).

Active Rac1 associates with membranes and promotes cell migration [37, 38, 39], and POTEE, Rac1 and actin filaments were visualized at the leading edge of HCC‐1569 cells, where Rac1 is activated (Fig. 2C,D). Indeed, POTEE localized selectively in the cytoplasm and in the membrane ruffles, structures formed in cells that migrate (Fig. 2B,C). Moreover, the cell lines MDA‐MB‐231 and HCC‐1569, which had higher levels of POTEE expression, demonstrated more active Rac1 and increased migratory capabilities compared to T‐47D, which had lower levels of POTEE expression (Fig. S1B,C). These results suggest that POTEE may play a role in influencing the migration capacity of breast cancer cells.

As expected, POTEE overexpression enhanced the migration and invasion of the triple‐negative MDA‐MB‐231 breast cancer cells. (Fig. 2E,F and Fig. S1D). Rac1 activation is required for cell migration [37, 40] and POTEE bound to Rac1 in cells exposed to HGF. Thus, we wondered if the enhanced cell migration in response to high levels of POTEE was due to the activation of this GTPase. POTEE overexpression augmented Rac1 activity (Rac1‐GTP bound) and hence, the migration and invasive capacity of the cells (Fig. 2G,H). Exposing BC cells to a specific Rac1‐inhibitor that reduce Rac1 activity (Fig. S1E) weakened their invasive capacity when POTEE was overexpressed (Fig. 2I), suggesting that the enhanced BC invasion was Rac1‐dependent.

Rac1 has also been implicated in tumor cell proliferation [13] and indeed, POTEE overexpression increased the rate of TNBC cell proliferation (Fig. 2J). Similar results were obtained when proliferation was assessed through the expression of the Ki67 marker in cells expressing high levels of POTEE (Fig. 2K,L).

All these results suggest that strong expression of POTEE enhances the tumorigenic capacity of TNBC cells by augmenting Rac1 activity.

### 3.3 The tumorigenic capacity of TNBC cells driven by POTEE requires Rac1‐SUMOylation

**Fig. 3:**
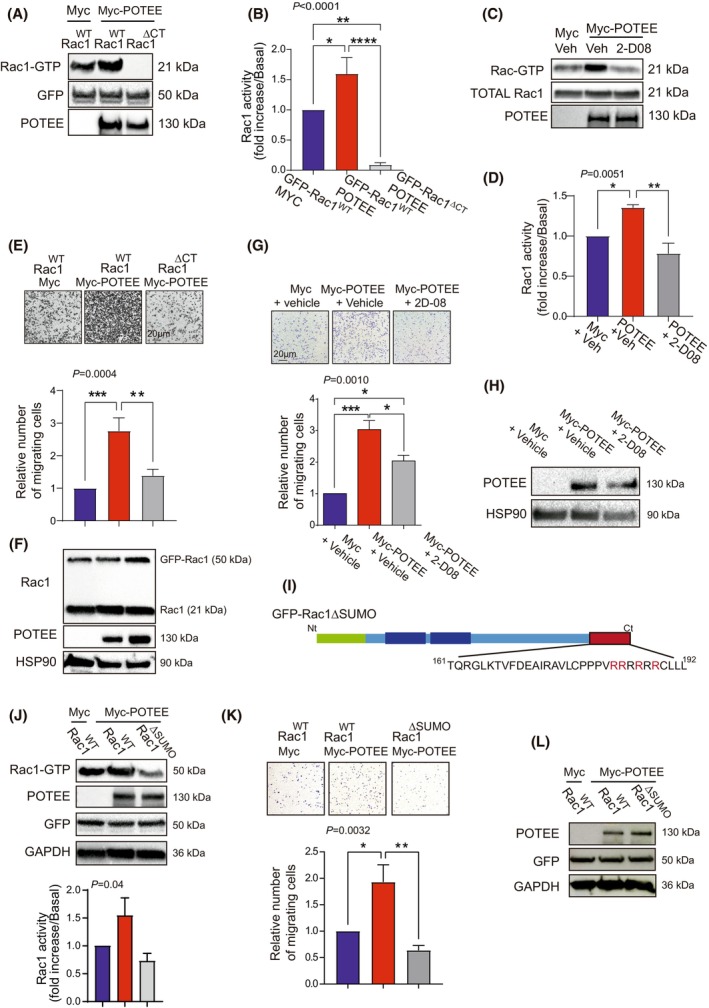
Rac1 SUMOylation is required for POTEE to regulate cell migration. (A) HEK293T cells transfected with plasmids containing the GFP‐Rac1WT or GFP‐Rac1∆CT and Myc‐POTEE constructs. Rac1 activity was assayed and Rac1‐GTP detected in western blots. (B) The relative normalized amounts of Rac1‐GTP in A were quantified by scanning densitometry (ANOVA test; Tukey's *post‐hoc* test). The results represent three independent experiments presented as mean ± SEM. (C) Myc epitope‐tagged POTEE was expressed in MDA‐MB‐231 cells maintained in the presence or absence of the SUMO inhibitor 2‐D08 (20 μm). The Rac1 activity was then assayed and Rac1‐GTP was detected by western blots. (D) The relative normalized amounts of Rac1‐GTP in C quantified by scanning densitometry. The results represent three independent experiments presented as mean ± SEM (ANOVA test; Tukey's *post‐hoc* test). (E) MDA‐MB‐231 cells transfected with plasmids containing the GFP‐Rac1WT or GFP‐Rac1∆CT and Myc‐POTEE constructs, the migration of which was assessed in transwell assays as seen in images of migrated TNBC cells at 24 h. Stained migrated cancer cells quantified as the number of migrating MDA‐MB‐231 cells relative to the controls. Scale bar, 20 μm. The results represent three independent experiments presented as mean ± SEM (ANOVA test; Tukey's *post‐hoc* test). (F) Representative blot of the cell transfection used in E is shown. (G) Migration assay from MDA‐MB‐231 cells transfected with Myc‐POTEE with or without the SUMO inhibitor 2‐D08 (20 μm). Stained migrated cancer cells quantified as the number of migrating MDA‐MB‐231 cells relative to the controls. Scale bar, 20 μm. The results represent three independent experiments presented as mean ± SEM (ANOVA test; Tukey's *post‐hoc* test). (H) Representative blot of the cell transfection from G is shown. (I) Representative scheme of the Rac1 form used in the experiments. GFP (green) is fused to the N‐terminal (Nt) of the Rac1 protein (light blue bar). Mutation of the SUMO lysines to arginines (shown in red) present in the C‐terminal (Ct) region results in the mutant form of Rac1: GFP‐Rac1ΔSUMO. (J) MDA‐MB‐231 cells were transfected with plasmids containing the GFP‐Rac1WT or GFP‐Rac1∆SUMO and Myc‐POTEE constructs, assaying Rac1 activity and Rac1‐GTP was detected by western blots. The results represent three independent experiments presented as mean ± SEM (ANOVA test; Tukey's *post‐hoc* test). (K) Transwell migration assays and images of migrated MDA‐MB‐231 cells at 24 h. Migrated cancer cells quantified as the number of migrating cells relative to the controls (ANOVA test, Tukey's *post‐hoc* test). (L) A representative blot of the cell transfection from J is shown. The results represent three independent experiments presented as mean ± SEM. Significant differences were assessed and considered when *P* < 0.05 (**** < 0.0001, *** < 0.001, ** < 0.01, * < 0.05). When *P* > 0.05, differences were considered non significative (ns).

To analyze whether Rac1 SUMOylation was required for the tumorigenic activity of POTEE, we transfected HEK293T cells with plasmids expressing GFP‐Rac1 WT or a truncated Rac1 that cannot be SUMOylated and does not interact with POTEE (GFP‐Rac1∆CT). The activity of Rac1‐GTP in the presence of high levels of POTEE fell when Rac1 could not be SUMOylated (Fig. 3A,B) and similar results were obtained when TNBC cells were exposed to 2‐D08, an inhibitor of the SUMO machinery (Fig. 3C,D).

To confirm that POTEE induced migration through SUMOylated Rac1, we transfected TNBC cells with GFP‐Rac1WT or GFP‐Rac1∆CT that does not interact with POTEE. In the presence of high levels of POTEE, the enhanced migratory capacity of TNBC cells required the ability of Rac1 to interact with POTEE (Fig. 3E,F). Moreover, inhibiting the SUMOylation machinery with 2‐D08 dampened the migration observed in the presence of high levels of POTEE (Fig. 3G,H).

Rac1 SUMOylation occurs at any of the lysine residues in the C‐terminal PBR [14] (Fig. 3I). As expected, a GFP–Rac1 mutant in which the four lysines in the PBR were replaced with arginines (GFP–Rac1ΔSUMO) was sufficient to dampen Rac1 activation and cell migration in the presence of POTEE (Fig. 3J–L).

Overall, these results indicate that the tumor cell migration/invasion induced by POTEE requires Rac1 SUMOylation.

### 3.4 Rac1 is required for POTEE‐induced invadopodium formation

The Rac1 GTPase play critical roles in coordinating the force generated by the formation of cellular protrusions, as well as cell–cell and cell–matrix adhesions. Thus, Rac1 stimulates the formation of a protrusive leading edge named lamellipodia required for cell migration and it drives the trafficking of MMPs to the tips of invadopodia in order to promote cancer cell metastasis [6, 7, 41].

**Fig. 4:**
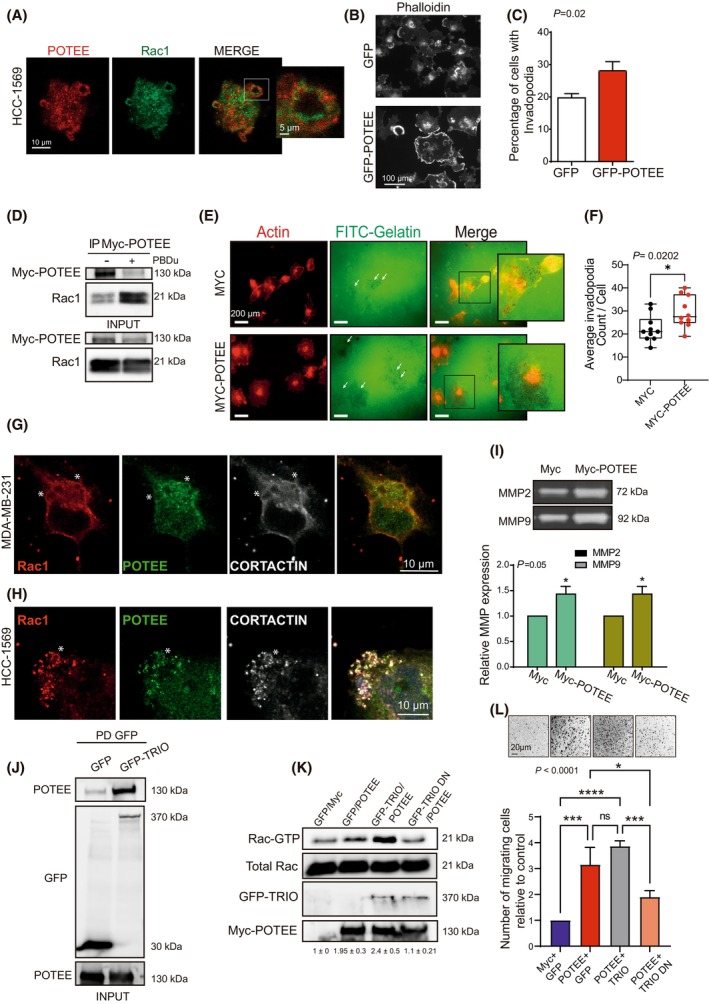
POTEE induces invadopodia formation by activating non‐SUMOylated Rac1. (A) Representative images of endogenous POTEE and Rac1 in invadopodia‐like structures in HCC‐1569 cells. Scale bar, 10 μm. (B) Representative images of MDA‐MB‐231 cells expressing GFP or GFP‐POTEE, treated with phorbol esters (PBDu) for 30 min and stained for phalloidin. Scale bar, 100 μm. (C) The proportion of cells from group B forming invadopodia was determined by counting 100 cells per experiment. The results presented here represent the mean ± SEM of three independent experiments, and statistical analysis was performed using the *χ* ^2^ test. (D) MDA‐MB‐231 cells transfected with Myc‐POTEE and treated with PBDu (Phorbol 12, 13‐dibutyrate), from which POTEE was immunoprecipitated (IP) and Rac1 was detected in western blots. (E) Representative images showing gelatine degradation by invadopodia in response to POTEE expression. Scale bar, 200 μm. Arrows indicate the region of gelatine degradation. (F) Quantification of the number of invadopodium per cell from E. The dots on the graph represent the number of invadopodium per analyzed cell. (*t*‐test). (G, H) Immunofluorescence images of triple‐negative breast cancer (TNBC) MDA‐MB‐231 (G) and HER2+ HCC‐1569 (H). The asterisks indicate the structure of invadopodia. Endogenous Rac1 (red), POTEE (green) and Cortactin (white) co‐localize at invadopodia‐like protrusion structures. Invadopodia were observed in cells as rosettes after PBDu treatment to induce them. Scale bar 10 μm. (I) Representative zymogram gels corresponding to the MMP‐2 and MMP‐9 (Metalloproteinases 2 and 9, respectively) degradation bands. Gelatine zymography of the bands in F quantified by scanning densitometry. The results represent three independent experiments presented as mean ± SEM (2‐way ANOVA test, Sidak's *post‐hoc* test). (J) HEK293T cells were transfected with GFP or GFP‐TRIO, POTEE was immunoprecipitated and GFP was detected in western blots. (K) Rac1 activity was assayed in HEK293T cells transfected as shown and Rac1‐GTP was detected in western blots. The numbers indicated below the image represent the quantification of Rac1 activity from two independent experiments ± SD. (L) Transwell migration assays performed on MDA‐MB‐231 cells transfected with plasmids carrying GFP‐TRIO or GFP‐TRIO DN (dominant negative mutant) and Myc‐POTEE constructs, and images of migrated triple‐negative breast cancer cells (MDA‐MB‐231) at 24 h. Scale bar 20 μm. The number of migrating MDA‐MB‐231 cells relative to the controls. The results represent three independent experiments presented as mean ± SEM (ANOVA test, Tukey's *post‐hoc* test). Significant differences were assessed and considered when *P* < 0.05 (**** < 0.0001, *** < 0.001, * < 0.05). When *P* > 0.05, differences were considered non significative (ns).

Overexpression of POTEE in TNBC cells induces the formation of protrusions that resemble invadopodia (Fig. S2A). Additionally, the co‐localization of endogenous Rac1 and POTEE was observed in these protrusions (Fig. 4A). Thus, we wondered if the Rac1/POTEE interaction was required to efficiently assemble cytoskeletal structures involved in invasion like invadopodia. As such, the ability of BC cells overexpressing POTEE to form invadopodia was assessed in response to short‐term exposure to phorbol esters (PBDu), a treatment widely used to stimulate invadopodia and podosome assembly by cells [42]. POTEE overexpression induced the formation of more invadopodia than in the control cells (Fig. 4B,C) and POTEE interacted more efficiently with Rac1 in response to PBDu (Fig. 4D). Invadopodia are often found in association with sites of extracellular matrix (ECM) degradation. To investigate the impact of POTEE on ECM degradation, we utilized a gelatin‐based invadopodia assay. MDA‐MB‐231 cells were seeded onto slides coated with fluorescent (FITC) gelatin. Staining for F‐actin revealed that the overexpression of POTEE increased the number of invadopodia compared to the control condition. Importantly, these invadopodia structures were observed to coincide with areas of gelatin degradation, which appeared as dark regions (Fig. 4E,F and Fig. S2B).

Moreover, POTEE and Rac1 localized with Cortactin in the invadopodia of HCC‐1569 and MDA‐MB‐231 cells (Fig. 4G,H).

The invadopodium is a specialized subcellular actin‐rich structure formed by cancer cells and that is involved in matrix degradation. Thus, we wondered if POTEE overexpression might enhance the secretion of some MMPs involved in matrix degradation by cancer cells. Indeed, two of the main MMPs secreted in BC, MMP2 and MMP9, were enhanced in response to high levels of POTEE (Fig. 4I).

The interaction of POTEE with Rac1‐SUMO1 induced Rac1 activation (Figs 2 and 3) and two of the Rac1 GEFs known to participate in invadopodia formation are β‐PIX and TRIO‐GEF [6, 43]. β‐PIX interacts with Rac1 at its C‐terminal and it is not required for Rac1 activation by POTEE (Fig. S2C). By contrast, TRIO does interact with POTEE (Fig. 4J) and co‐localizes with POTEE in the invadopodia (Fig. S2D). Moreover, its overexpression, in combination with high levels of POTEE, induced Rac1 activation, an effect that was not produced by the dominant negative TRIO‐GEF (Fig. 4K). Similarly, the migratory capacity of TNBC MDA‐MB‐231 cells increased when POTEE was overexpressed but not in the presence of the dominant negative TRIO‐GEF (Fig. 4L). Furthermore, the increase in Rac1 activity mediated by TRIO and POTEE was abolished when the region of Rac1 that is SUMOylated was depleted (Fig. S2E).

### 3.5 Overexpression of POTEE increases the tumorigenic activity of breast cancer cells *in vivo*

**Fig. 5:**
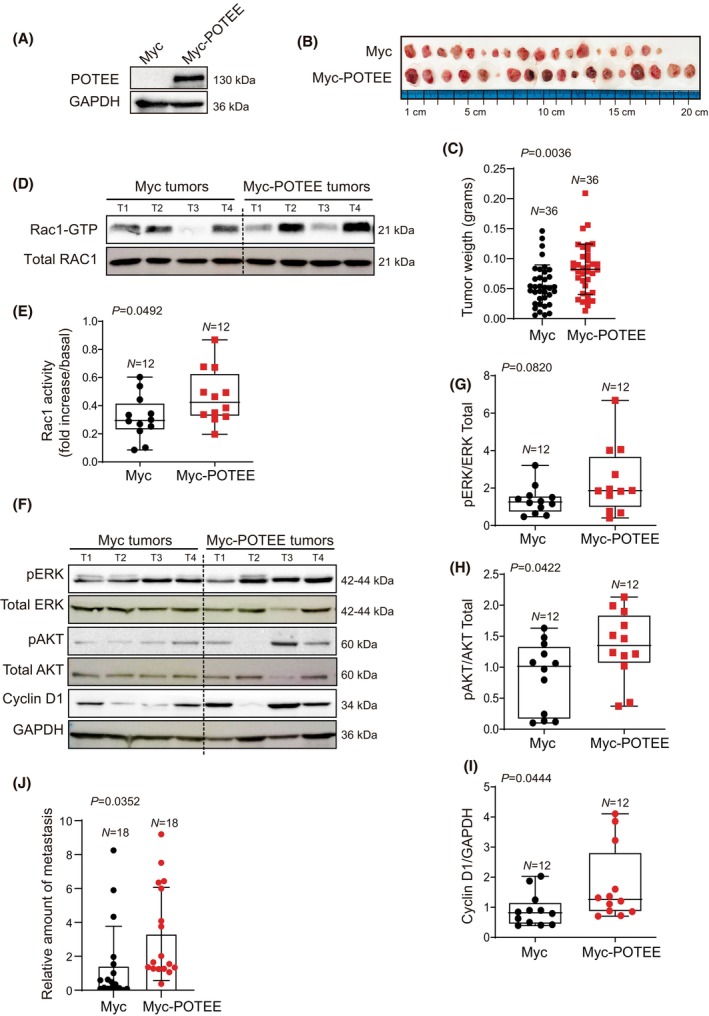
POTEE promotes tumor proliferation and metastasis *in vivo* by activating Rac1. (A) Western blot of POTEE expression in the MDA‐MB‐231 cells used for the *in vivo* experiment. (B) Representative images of tumors obtained from xenografts 7 days after inoculation. Scale bar centimeter (cm). (C) Tumor weight after inoculation of the CAM. Statistics were analyzed using an unpaired two‐tailed Student's *t*‐test. (D) Representative immunoblot of Rac1 activity in tumors from the xenograft. T1 to T4 are tumors derived from the same condition. (E) The Rac1‐GTP bands were quantified, and the normalized intensities were calculated relative to the controls (*t*‐test) (F) Representatives immunoblot of AKT and ERK activity or Cyclin D1 expression in tumors from the xenograft. Each number on top of the image (T1 to T4) represents an individual tumor. (G–I) The pERK/ERK (G), pAKT/AKT (H), Cyclin D1 (I) protein from F was quantified and the normalized intensities were calculated relative to the controls (*t‐*test). (J) Quantification of the MDA‐MB‐231 cells overexpressing POTEE in the bone marrow using the Alu sequences (*t‐*test). Data are presented as mean ± SD, and each dot represents an independent animal. Significant differences were assessed and considered when *P* < 0.05.

To investigate the significance of our discoveries *in vivo*, we introduced MDA‐MB‐231 cells expressing either Myc or Myc‐POTEE onto the CAM of 11‐day‐old chicken embryos. We validated that POTEE expression was stable for a minimum of 5 days (Fig. 5A). Subsequently, we explored the role of POTEE overexpression in tumor initiation and dissemination. Consistent with our *in vitro* findings, tumors originating from MDA‐MB‐231 cells with POTEE overexpression demonstrated larger size and mass compared to the control conditions (Fig. 5B,C).

To corroborate the implication of the POTEE‐Rac1 GTPase axis in tumor development, we investigated whether POTEE overexpression affected Rac1 GTPase activation in MDA‐MB‐231 cell‐derived tumors, and tumor cell dissemination. Similar to the results observed *in vitro*, POTEE was found to increase the activation of this GTPase (Fig. 5D,E) and aberrant POTEE/Rac1 tumor activation affected the activity of the downstream AKT and ERK signaling pathways (Fig. 5F–H), both of which act anomalously in cancer. Because Cyclin D1 is essential for breast cancer cell proliferation, we also evaluated its expression in these tumors and its expression was enhanced in tumors when POTEE was overexpressed (Fig. 5I).

We conducted PCR amplification of human specific Alu sequences to identify disseminated tumor cells in chicken bone marrow tissue [44]. We found a relative increase in metastasis when POTEE was overexpressed in the triple‐negative breast cancer cells, MDA‐MB‐231 (Fig. 5J).

All these results further suggest that POTEE expression promotes tumor proliferation and metastasis *in vivo* through Rac1 activation.

### 3.6 Rac1 co‐localizes with POTEE in aggressive breast tumors

**Fig. 6:**
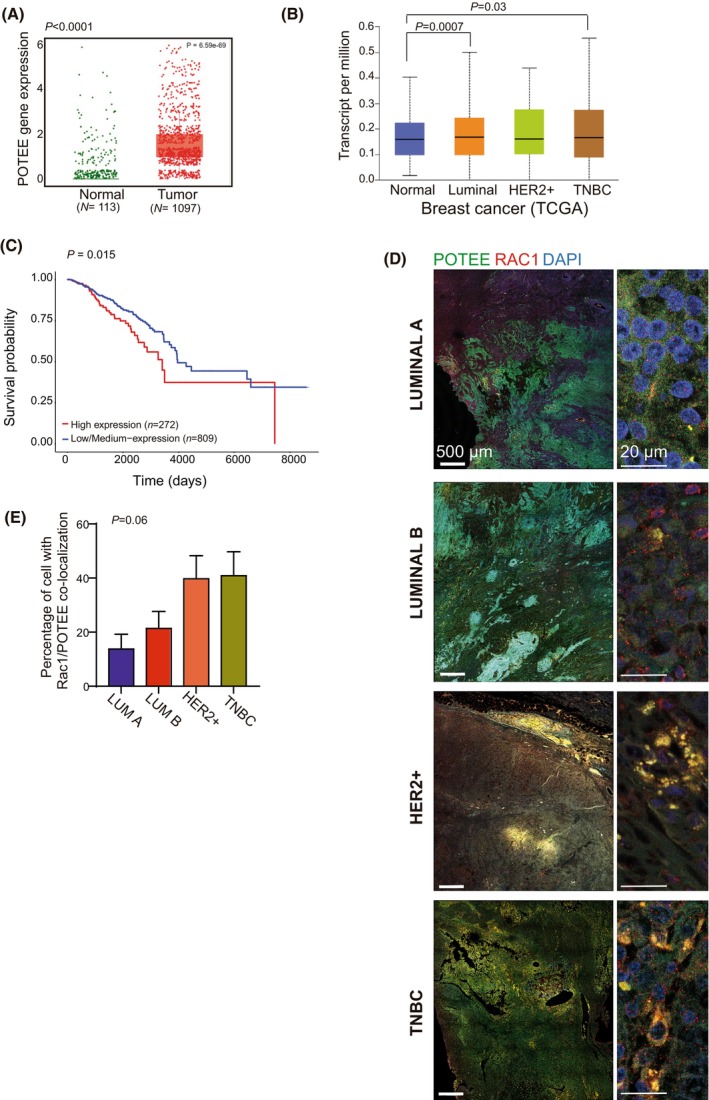
POTEE and Rac1 co‐localize in human tumor samples from more aggressive molecular subtypes. (A, B) *In silico* analysis reveals an elevated expression of POTEE in tumor samples compared to normal tissue (*t*‐test), as well as in various breast cancer subtypes (*t*‐test). The graphs were directly obtained from the UALCAN website. The datasets analyzed were sourced from TCGA. (C) KM survival analysis showing that stronger POTEE expression is associated with worse patient survival. Patients grouped according to POTEE protein expression. We used the KM estimator and log‐rank test to compare temporal variables. (D) Representative images of POTEE and Rac1 staining in human breast tumors of different molecular subtypes. Scale bar 500 μm. The right panel displays an illustrative image depicting the co‐localization of Rac1 and POTEE. Scale bar 20 μm. Four breast cancer tissues were analyzed for each molecular subtype, totalling four samples per subtype (*N* = 4). (E) Quantification of the proportion of tumor cells in which Rac1 and POTEE co‐localize (ANOVA test). *N* = 4. Data in bar graphs is expressed as the mean ± SD and analyzed by ANOVA. Significant differences were assessed and considered when *P* < 0.05.

*POTEE* accumulation was enhanced in invasive ductal breast carcinomas relative to normal mammary tissues (Fig. 6A). Indeed, *POTEE* was more strongly expressed in Luminal BC and TNBC subtypes when evaluated in TCGA datasets [45] (Fig. 6B). Interestingly, we did identify patients with poor BC evolution that express high levels of *POTEE* (Fig. 6C).

Having observed that Rac1 and POTEE are required for tumor cell invasion and invadopodia formation, we assessed whether Rac1 and POTEE co‐localization in samples from different molecular tumor subtypes might be related to the aggressive nature of tumors. We found that different molecular subtypes of breast tumors expressed high levels of Rac1 and POTEE (Fig. 6D), yet Rac1/POTEE co‐localized in a higher proportion of cells in aggressive tumor types like HER2+ and TNBC than in cells from tumors with a better prognosis (Fig. 6E). Interestingly, both proteins were expressed to a similar extent in the different tumors, but their co‐localization differed (Fig. S3A,B). Hence, these two proteins may induce a more aggressive phenotype in a specific context, in which the tumor cells induce stronger malignancy.

**Fig. 7:**
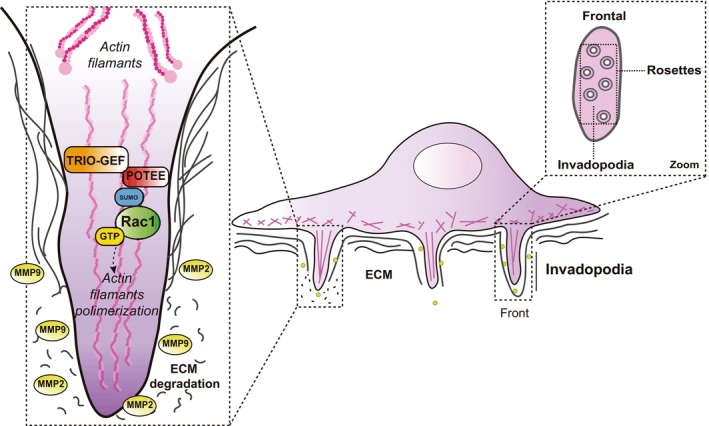
Working model of the mechanisms by which POTEE can regulate the invadopodia formation. POTEE interacts with Rac1 and promotes its activation by recruiting TRIO‐GEF in the invadopodia, thereby favoring tumor cell dissemination. ECM, extracellular matrix; MMP2 and 9, Matrix metalloproteases 2 and 9, respectively; Rac1‐GDP, the inactive form of Rac1 (Rac1 bound to guanosine diphosphate); Rac1‐GTP, the active form of Rac1 (Rac1 bound to guanosine triphosphate); TRIO‐GEF, TRIO‐guanine nucleotide exchange factor.

All the results indicate that POTEE contributes to the malignancy of breast cancer by interacting with SUMOylated Rac1 in the invadopodia, inducing Rac1 activation through the recruitment of TRIO‐GEF (Fig. 7).

## 4 Discussion

The role of POTEE in cancer has gained much interest in recent years as it has been seen to be overexpressed in various tumors, including colorectal, pancreatic and ovarian tumors [21, 22, 23, 24, 25]. Indeed, it has been associated with different events related to tumor development, such as proliferation, survival, and dissemination [23, 24, 25, 46]. Notably, recent evidence established POTEE mutations as a predictive marker in lung adenocarcinoma [47]. Furthermore, *in silico* studies indicated that POTEE, along with other members of the POTE family, could be suitable targets for anti‐cancer compounds, making it a potential therapeutic target [48].

Here, we discovered a novel role of POTEE in promoting BC dissemination, participating in the formation of invadopodia and interacting with Rac1‐SUMO1. While a possible association between POTEE and Rac1 has been proposed previously [24], our study not only confirms this interaction but also reveals a new interaction between POTEE and Rac1‐SUMO1. These findings suggest a previously unknown role of POTEE in the context of this PTM. Our data indicate that POTEE predominantly associates with the inactive form of Rac1‐SUMO1 and activates it, thereby enhancing tumor cell migration and invasion. Furthermore, POTEE participates in the formation of the invadopodia that facilitate cell invasion by degrading the ECM through the secretion of MMPs. POTEE overexpression increases MMP secretion and the relative number of invadopodia in response to PBDu, which stimulates the assembly of these actin‐rich protrusions. Rac1 may influence invadopodia dynamics when activated by the GEF TRIO [6]. Accordingly, our results demonstrate an interaction between POTEE and TRIO that is essential for POTEE to promote migration/invasion. Therefore, POTEE provides a platform that brings together GDP‐bound Rac1 and its GEF TRIO, promoting Rac1 activity within invadopodia to in turn facilitate tumor invasion. However, it is important to note that the interaction between POTEE and other GEFs cannot be completely ruled out. Our observations indicate that POTEE co‐localizes with Rac1 at the membrane ruffles, suggesting its potential role in promoting migration by influencing the formation of these structures. Moreover, we found that inhibiting Rac1 activity reduces the migration capacity of MDA‐MB‐231 cells with overexpression of POTEE. It is known that at the concentration used in this study, the inhibitor EHop‐016 reduces the association of Rac1 with other GEFs, such as VAV2. [49]. Therefore, it is plausible that GEFs such as VAV2 is involved in the activation of Rac1 in response to POTEE, regardless of the SUMOylation state of Rac1.

Significantly, POTEE has previously been defined as a scaffold protein, particularly in the context of mTORC2 activation. POTEE can interact with mTOR and RICTOR to trigger mTORC2 signaling, potentially through its ankyrin domains [46, 50]. Interestingly, TRIO interacts with the ANKR26 protein that also contains such ankyrin domains [51], further supporting the need to explore the specific domains of POTEE involved in its interaction with TRIO.

The effects of POTEE on Rac1 activity and invasion were further validated using the CAM model, and by detecting elevated levels of POTEE in the most aggressive BC cell lines. As occurs in the TNBC cell line, POTEE overexpression enhances cell proliferation and viability, as witnessed by the increase in tumor weight, AKT phosphorylation and Cyclin‐D expression. These molecular events could be a direct consequence of Rac1 activation, as Rac1 is thought to activate AKT signaling, also promoting NF‐κB activation by inducing PI3K [52]. Given that POTEE expression has also been associated with AKT activation [25, 50], it would be interesting to study the POTEE/Rac1 axis in this context. Indeed, an increase in Cyclin‐D may also be induced by Rac1 activation through its stimulation of NF‐κB [53].

Rac1 SUMOylation, a PTM that occurs in response to an EMT stimulus, is essential for correct tumor cell migration while not affecting other cellular processes [14, 17]. Therefore, it will be of particular interest to explore the involvement of POTEE in this Rac1 PTM. Our findings demonstrate that POTEE localizes to the membrane ruffles where Rac1 SUMOylation takes place, and they reveal that Rac1 SUMOylation is necessary for POTEE to influence migration and invasion. Besides, POTEE interacts with the C‐terminal region of Rac1 where SUMO attaches, adding POTEE to the extensive list of Rac1 interactors that bind to this specific region [54]. This finding, together with the strengthened interaction observed between POTEE and Rac1‐SUMO1, suggest a possible association between POTEE and the SUMO peptide itself that might be explored in future studies. Notably, POTEE contains potential SIM sites within its sequence.

Furthermore, it is important to note that POTEE co‐localizes with Rac1 in membrane ruffles. Our study has demonstrated that overexpressing POTEE leads to a reduction in migration and invasion through the formation of invadopodia, which is dependent on Rac1‐SUMO1 signaling. However, we believe that POTEE may also potentially contribute to migration through its involvement in promoting membrane ruffle formation. This aspect of POTEE's role in migration will be explored in future studies.

Overall, our work highlights the role of POTEE and Rac1 in tumor dissemination. It is important to note that it is not solely the expression of POTEE or Rac1 alone but rather their co‐localization that determines the aggressiveness of BC tumors, as demonstrated by analyzing patient samples. This highlights the crucial role of Rac1 regulation in tumor progression. Moreover, our public database analysis provides evidence that POTEE expression is associated with poor BC evolution, further evidence of its involvement in tumor progression. In this regard, it would indeed be interesting to genetically deplete POTEE to evaluate its potential as a therapeutic strategy for reducing the dissemination of metastatic breast cancer.

## 5 Conclusions

Our results indicate that POTEE contributes to tumor malignancy by activating Rac1, which must be SUMOylated to promote tumor dissemination. Given its role in tumor invasion, this reinforces the idea of POTEE as a potential therapeutic target, the leading cause of cancer‐related deaths.

## Conflict of interest

The authors declare no conflict of interest.

## Author contributions

Performing the experimental and data analysis: SC‐L, AM‐L, AG‐C, GI, MG‐F, NS, ML, MM‐E; Conceptualization of the study: SC‐L, AM; interpretation of human samples: SG‐M, PV‐C, GV; critically reviewed the manuscript: GV, AM; provided team leadership, funding acquisition, project management and wrote the manuscript: SC‐L.

### Peer review

The peer review history for this article is available at https://www.webofscience.com/api/gateway/wos/peer‐review/10.1002/1878‐0261.13568.

## Supporting information


**Fig. S1.** Rac1 SUMOylation and activity in breast cancer cells.
**Fig. S2.** POTEE is required for invadopodia formation.
**Fig. S3.** Localization analysis of POTEE and Rac1 proteins in tumor samples from breast cancer patients.

## Data Availability

The datasets obtained and/or analyzed in the current study are available from the corresponding author upon reasonable request.
