# Supplementary material for: POTEE promotes breast cancer cell malignancy by inducing invadopodia formation through the activation of SUMOylated Rac1
Source: Mol Oncol. 2023 Dec 27;18(3):620–40. doi: 10.1002/1878-0261.13568 (PMC10920093; doi:10.1002/1878-0261.13568)
Supplement: Supplementary file 1 — Fig. S1. Rac1 SUMOylation and activity in breast cancer cells. Fig. S2. POTEE is required for invadopodia formation. Fig. S3. Localization analysis of POTEE and Rac1 proteins in tumor samples from breast cancer patients. [file MOL2-18-620-s001.pdf]

POTEE promotes breast cancer cell malignancy by inducing invadopodia formation through the activation of SUMOylated Rac1

Angélica Martínez-López[1,2#], Ana García-Casas[1,2#], Guiomar Infante[1,2], Mónica González-Fernández[1,2], Nélida Salvador[1,2], Mar Lorente[1,2], Marina Mendiburu-Eliçabe[3], Santiago Gonzalez-Moreno[4], Pedro Villarejo-Campos[5], Guillermo Velasco[1,2], Angeliki Malliri[6] and Sonia Castillo-Lluva[1,2]*

[1]*Departamento de Bioquímica y Biología Molecular, Facultad de Ciencias Químicas, Universidad Complutense, Madrid 28040, España.*

[2]*Instituto de Investigaciones Sanitarias San Carlos (IdISSC), Madrid 28040, España.*

[3]*Departamento de Estadística e Investigación Operativa, Facultad de Ciencias Matemáticas, Universidad Complutense de Madrid, Madrid 28040, España.*

[4]*MD Anderson Cancer Center Madrid, Madrid 28033, España.*

[5]*Hospital Universitario Fundación Jiménez Díaz, Madrid 28040, España.*

[6]*Cancer Research UK Manchester Institute, The University of Manchester, M20 4BX Manchester, UK.*

([#]) Equal contribution as first authors.

([*]) Corresponding author: Sonia Castillo-Lluva, Departamento de Bioquímica y Biología Molecular, Facultad de Ciencias Químicas, Universidad Complutense, Madrid, España. Phone: +34 91 3944276; FAX: +34 91 3944872; E-mail: sonica01@ucm.es.

Figure S1. Rac1 SUMOylation and activity in breast cancer cells. A) SUMOylation of Rac1 GTP in different breast cancer cell lines representing different molecular subtypes. **B**) Representative western blot of active Rac1 in different breast cancer cell lines Quantification of the relative normalized amounts of Rac1 GTP, as determined by scanning densitometry from at least three independent experiments using ImageJ software (ANOVA test; Tukey's post hoc test). **C**) Transwell migration assays showing the number of migrating HCC 1569 breast cancer cells relative to T 47D (t test). **D**) POTEE mRNA levels in MDA MB 231 cells transfected with Myc tag and Myc POTEE were compared to endogenous levels in HCC 1569 cells using RT qPCR. Relative quantification (RQ) was determined normalized to the control. **E**) Representative western blot of active Rac1 (Rac1 GTP) in the presence or absence of the Rac1 inhibitor (2 μM) relative to the control. The results represent three independent experiments presented as mean ± SEM. Significant differences were assessed and considered when $P<0.05$ (****<0.0001, ***<0.001, **<0.01, *<0.05). When $P>0.05$, differences were considered non significative (ns).

Figure S1

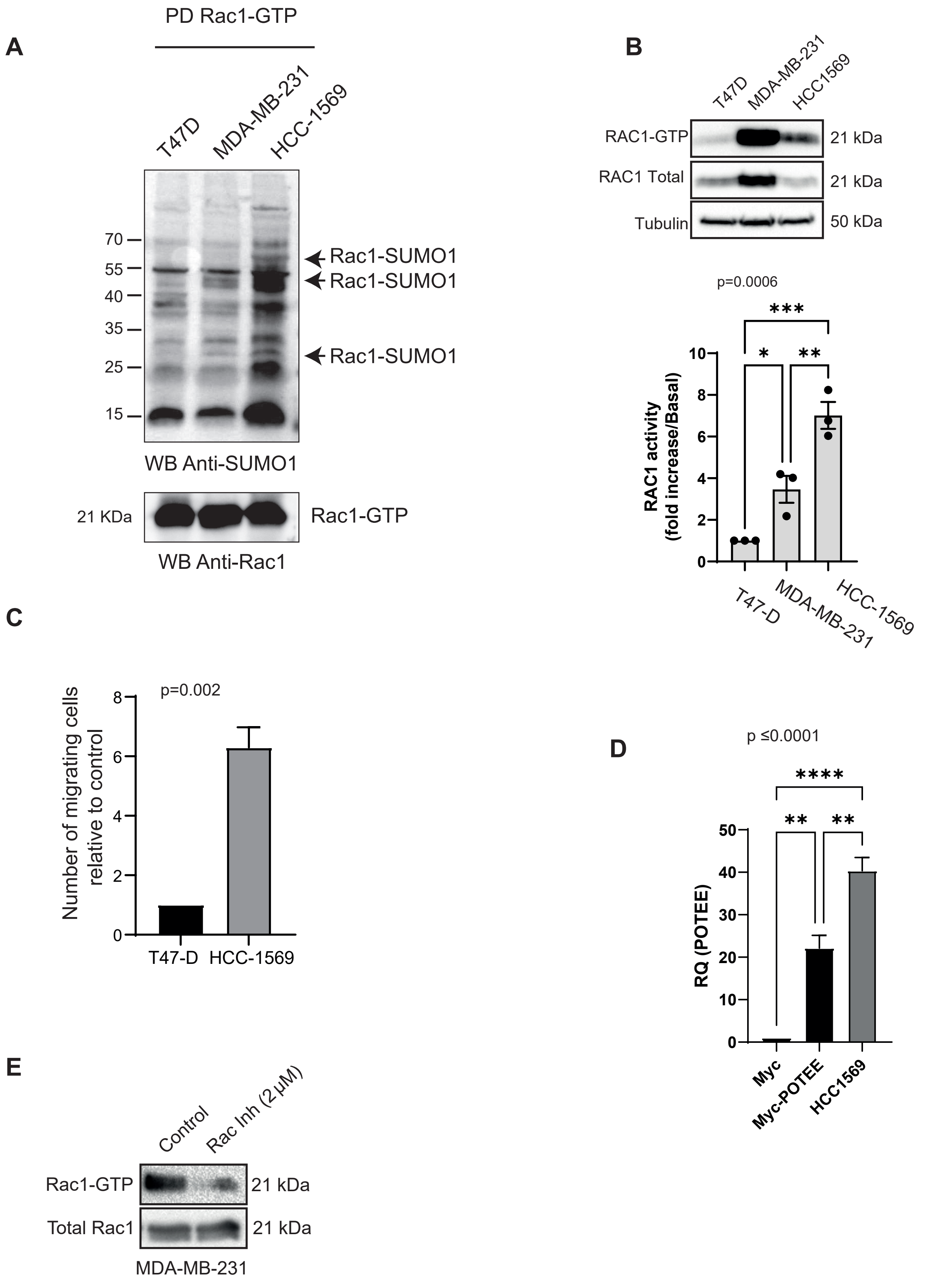
A
PD Rac1-GTP
T47D
MDA-MB-231
HCC-1569
70
55
40
35
25
15
Rac1-SUMO1
Rac1-SUMO1
Rac1-SUMO1
WB Anti-SUMO1
21 KDa
Rac1-GTP
WB Anti-Rac1
B
T47D
MDA-MB-231
HCC1569
RAC1-GTP
RAC1 Total
Tubulin
21 kDa
21 kDa
50 kDa
p=0.0006
***
*
**
RAC1 activity
(fold increase/Basal)
T47-D
MDA-MB-231
HCC-1569
C
p=0.002
Number of migrating cells
relative to control
T47-D
HCC-1569
D
p ≤0.0001
****
**
**
RQ (POTEE)
Myc
Myc-POTEE
HCC1569
E
Control
Rac Inh (2 µM)
Rac1-GTP
Total Rac1
21 kDa
21 kDa
MDA-MB-231

Figure S2. POTEE is required for invadopodia formation. (**A**) Representative images of GFP-POTEE localization in MDA-MB-231 cells. Scale bar, 100 μm. (**B**) Gelatine degradation assay was performed to assess the impact of POTEE overexpression on the ability of cells to form invadopodia and degrade gelatine. Scale bar 200μm. (**C**) Representative western blot of MDA-MB-231 cells probed for active Rac1 in response to POTEE and β-PIX overexpression. (**D**) Representative images of GFP-TRIO and Myc-POTEE in 293T cells co-localization in MDA-MB-231 cells. Arrows indicate the invadopodia co-localization. Scale bar, 100 μm. (**E**) Rac1 activity was assayed in HEK293T cells transfected as shown and Rac1-GTP was detected in western blots.

Figure S2

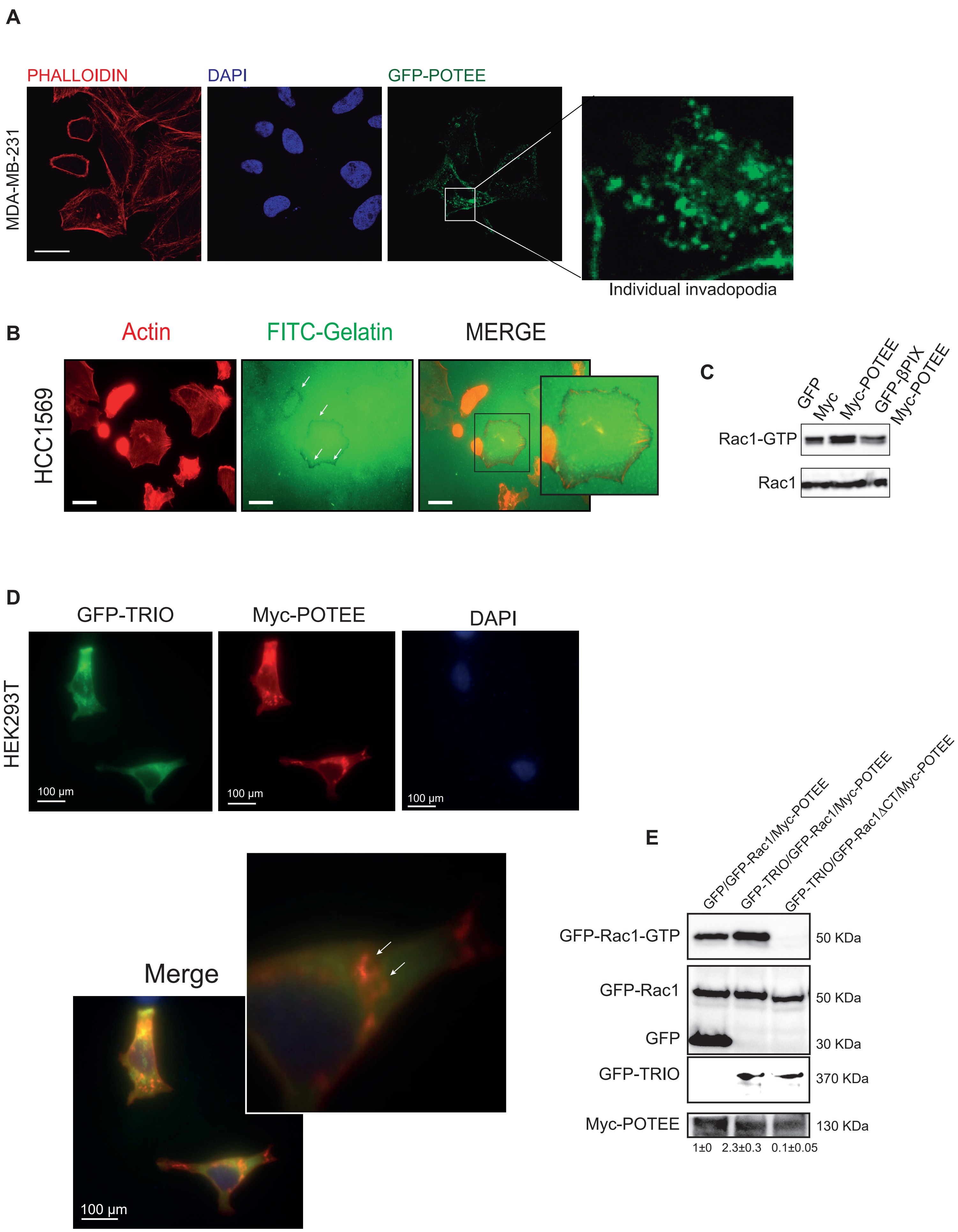
A
PHALLOIDIN
DAPI
GFP-POTEE
MDA-MB-231
Individual invadopodia
B
Actin
FITC-Gelatin
MERGE
HCC1569
C
GFP
Myc
Myc-POTEE
GFP-βPIX
Myc-POTEE
Rac1-GTP
Rac1
D
GFP-TRIO
Myc-POTEE
DAPI
HEK293T
100 μm
100 μm
100 μm
Merge
100 μm
E
GFP/GFP-Rac1/Myc-POTEE
GFP-TRIO/GFP-Rac1/Myc-POTEE
GFP-TRIO/GFP-Rac1ΔCT/Myc-POTEE
GFP-Rac1-GTP
50 KDa
GFP-Rac1
50 KDa
GFP
30 KDa
GFP-TRIO
370 KDa
Myc-POTEE
130 KDa
1±0
2.3±0.3
0.1±0.05

Figure S3. Localization analysis of POTEE and Rac1 in tumor samples from breast cancer patients. (**A**) Representative images of POTEE and Rac1 staining in human breast tumors of different molecular subtypes. (**B**) Quantification of Rac1 and POTEE positive cells in human breast tumor of different molecular subtypes.

Figure S3

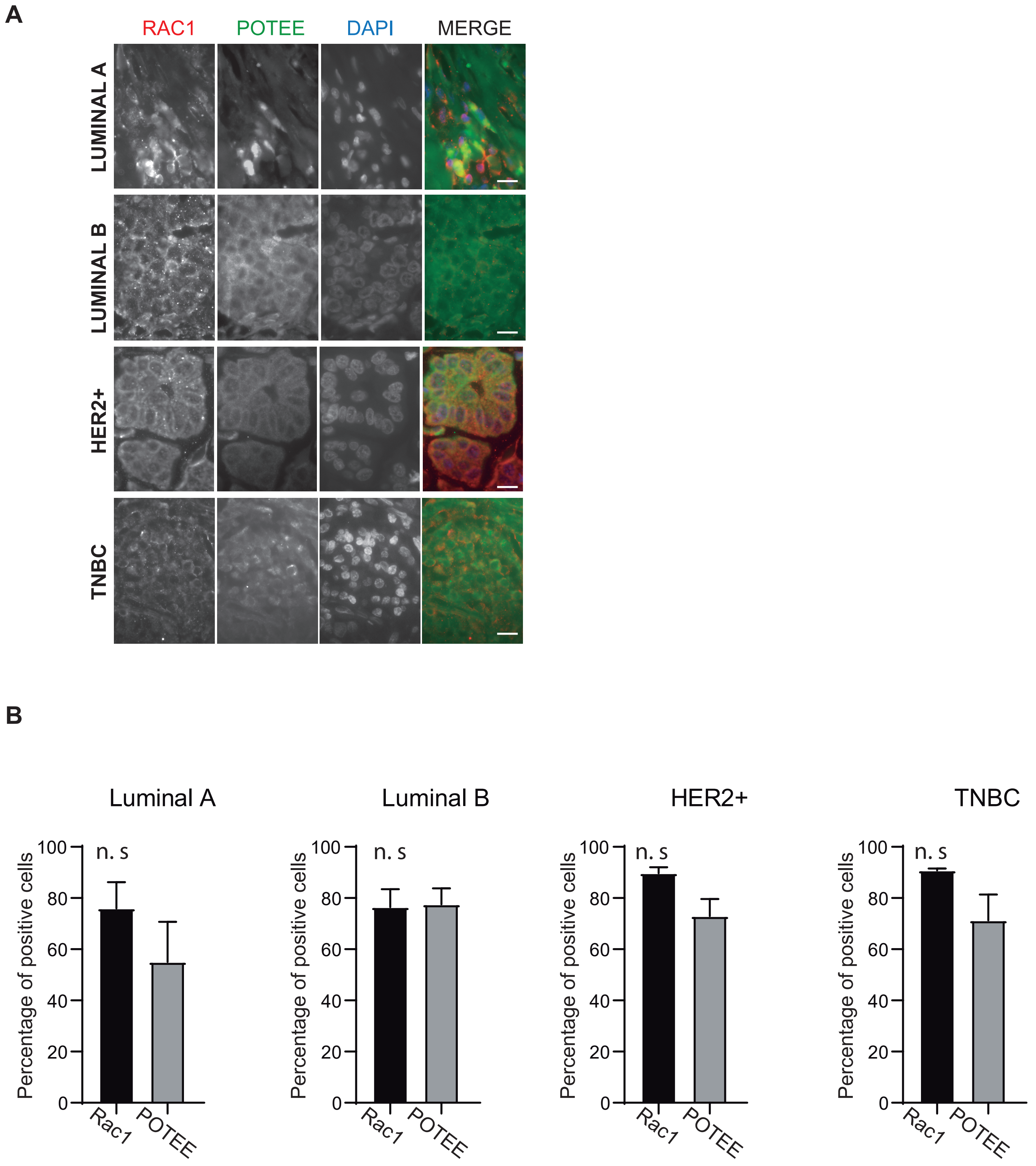
A
RAC1
POTEE
DAPI
MERGE
LUMINAL A
LUMINAL B
HER2+
TNBC
B
Luminal A
Luminal B
HER2+
TNBC
n. s
Percentage of positive cells
100
80
60
40
20
0
Rac1
POTEE
